# The Institutional Library

**Published:** 1919-03-01

**Authors:** 


### THE INSTITUTIONAL LIBRARY.

### CRIME AND CRIMINALS.*

IN the preface to an instructive and learned monograph entitled "The Rationale of Punishment," published in London not many years ago, the author, Dr. H. Oppenheimer, remarks that if an apology for his book was required he might well plead the meagreness of English literature on this subject. If "meagreness of literature" be taken to mean a small number of writers, it is undeniable, and perhaps not wholly regrettable, that in recent years at least the number of books and articles on the topics of crime, criminals, and punishment, in their various aspects, which has appeared in this country has been far less than that produced in the United States of America or in either France, Italy, or Germany. But it is none the less true that a large proportion of publications dealing with crime is very disappointing to the student who sets himself to the serious study of them, and that he will find himself too often involved in a mass of indefinite statements, equivocal terminology, and confused thinking.

Not only does the book now before us, like that which we have mentioned above, require no apology, but it is deserving of a warm welcome from all who may be interested in its subject-matter, and these are not few. Dr. Mercier is exceptionally well equipped for the task he has undertaken, for he is a well-known psychologist, and an experienced authority in mental pathology. He has, moreover, had the care of many criminals in asylums, and has made numerous examinations of the mental condition of persons, both sane and insane, who were charged with criminal offences. We think the careful reader of this book will find justification for the high praise which we attribute to the author for this production of one of the most thoughtful and clearly-written treatises on crime and criminals that has yet appeared.

At the present time it may be said that the chief contention between writers on crime and criminals turns on the long-posed question whether law-breakers are naturally disposed to crime, or are made criminals by the influences of the society in which they live. There is a school which attributes criminal action exclusively to "nature" and another, which goes to the extreme of almost wholly ignoring the natural element. Dr. Mercier attributes criminal conduct to two factors, the internal and the external, and holds that these factors are intimately, though differently, combined in every individual who commits crime, just as they are thus combined in non-criminal men. "Like all other action," he says, "criminal action is action whose ultimate purpose is dictated by instinct, and at the same time is guided by reason towards the attainment of its purpose." Crime, which is action injurious to society, is thus an incident in the social state, and the earliest step in the civilisation of the human criminal is when he learns from experience the necessity of some degree of forbearance and self-sacrifice. The doctrine that all men are potential criminals, held practically by all students of criminality, is a corollary of this position.

Following on the first chapter, "The Factors of Crime," the author treats of the "Psychology of Crime," and the "Nature of Crime." He continues with some highly instructive and practical chapters on the various kinds of crime and criminals, and concludes with important comments on prevention, detection, and punishment. One of the most useful lessons to be gathered from this book is that the proper study and treatment of criminals should be directed to the individual, not to the mass. Bearing this in mind, we would note a point of difference from Dr. Mercier in respect of a statement at the end of his book that the habitual criminal is beyond the reach of reform, and that the only criminal who can be reformed is the young criminal whose criminality is not as yet confirmed. To discuss this at any length would involve enquiry into what is meant by reform and what by confirmed criminality. We think that Dr. Mercier's classification of "habitual criminals" (see his pages 234-235) into two groups, viz. (1) those in whom criminality shows itself at a very early age—often called "instinctive" criminals; and (2) those who drift into crime, quasi-passively, as the course of least resistance, is quite sound and of great practical value. But mainly on the ground of experience of individual criminals, while at the same time doubting that any real value can be attributed to the term "confirmed criminality," we hold that many adult habitual criminals can be and are "reformed," in the same sense as the younger confessedly are "reformed," after a criminal career of many years and at ages far beyond youth. This is the only instance in Dr. Mercier's book which seems to us to illustrate the statement in his preface that the "deductive" method of reasoning is almost exclusively used by him; and we may say incidentally that with the apparent meaning he gives to the word "deductive" in this context or with consequent remarks on "deduction" and "induction" we cannot agree. Besides this one instance at the end of the book, there is, in our opinion, no reason whatever for Dr. Mercier to anticipate, as he says that he does, no greater welcome for his book than has been "accorded to other workers by the deductive method," except, perhaps, from a misguided few who skim the preface and read no more. We at least acquit Dr. Mercier of the self-incriminating charge of reasoning only by the method of deduction. At any rate, we have no doubt that this book will be placed in the first rank of all works on the subject it deals with. It has already been deemed worthy of the quinquennial award by the adjudicators of the "Swiney Prize" (who are conjointly the *Society of Arts* and the

* *Crime and Criminals.* By Charles Mercier, M.D., F.R.C.P., F.R.C.S. (University of London Press, 1918.)

**The Institutional Library**—(*continued*).

*Royal College of Physicians*) for the best recent work on medical or general jurisprudence. This is the second time that this valuable prize has fallen to Dr. Mercier, the former award having been made ten years ago for his well-known work on "Criminal Responsibility," which is an admirably fitting complement to his present treatise.

**Materia Medica and Therapeutics.** By J. Mitchell Bruce, M.A., LL.D., M.D., and Walter J. Dilling, M.B., Ch.B. Eleventh Edition. (London: Cassell and Co., Ltd. 1918. Pp. 675 + xiii. Price 9s. net.)

This book is well known to the medical profession. It has been read by many generations of students, and is still a popular manual among those who have yet to face the ordeal of professional examinations. Like others of its kind, it contains numerous details which the examiners demand but which, when this demand is satisfied, are of no further use to the practitioner. How far such books will survive the growing influence of pharmacology remains to be seen, but it is difficult to believe that the medical student in these days of reconstruction will continue to be pestered with detailed descriptions of the physical characters of kousso and cascarilla and euonymus bark. Important as such knowledge is to the manufacturing pharmacist, it is a mere dull mechanic exercise in the medical curriculum. In the meantime the examiners hold the field and the student must submit himself to their standards. The main part of the book, let it be gladly admitted, deals with the uses of drugs rather than with their properties, and it has been brought in successive editions to a highly creditable and satisfactory level. As an introduction to the treatment of disease a better guide can hardly be found, and the authors in their latest issue do not show any decline either in their ambitions or in their determination to achieve these. They boast the discussion of some forty new remedies, though of the ultimate fate of these they would probably be reluctant to give any guarantee; also sections dealing with electricity, massage, and exercise. Particular attention has been given to the developments in antiseptics and surgical dressings which we owe to the war. Other subjects of special interest are the authorised modifications in many of the official preparations arising out of the short supply of fats, sugar, glycerine, etc., and the stringent regulations governing the sale of cocaine and other alkaloids. These latter, be it noted, have a direct relation to the medical profession, for it is a legal offence to prescribe cocaine except in the form and under the conditions defined by the Order issued under the Defence of the Realm Act. We notice a slip on page 280 where "$\frac{1}{2}$ fl. dr." is written instead of $\frac{1}{2}$ fluid ounce.

**The Economic Basis of an Enduring Peace.** By C. W. Macfarlane, C.E., Ph.D. (Philadelphia: George W. Jacobs and Co. 1918. Pp. 79.)

This is a very interesting study of the contribution made by economic forces to the causation of the Great War. Moreover, it forms particularly appropriate reading at a time when wise men from all lands are gathered in conference, not only to make peace, but also to ensue it. Though Dr. Macfarlane holds most firmly the conviction that it is necessary for the welfare of the world to bring Germany's schemes to nought, he does not find that the war may be classified as a mere expression of moral obliquity or as an example of the superfluity of naughtiness. Rather he emphasises the economic pressure which prompted it. "The tragedy" (writing before the armistice) "is due," he says, "to the very *present* and pressing necessity to provide an adequate supply of iron and food to permit the growth of Germany in industry, wealth, and population." Coal and iron in abundance are the essentials of industrial success, and Dr. Macfarlane's argument is that this proposition will exercise a commanding influence on the stability of any new arrangement proposed in Europe. Unless each of the great competitors, France and Germany, is provided with a reasonable supply of the great sources of industrial wealth trouble may be anticipated. That Germany wanted more than this is evident enough, and she must be compelled to realise that her plans have been thwarted. But the Germans remain, many millions of them, and it is impossible to contemplate this mass of mankind in the heart of Europe and to believe that without reasonable employment they will be a centre of peace. Hence, so the argument runs, while the position of France in reference to supplies of coal and iron must be improved, Germany must not be reduced to helplessness. How this double demand can be satisfied Dr. Macfarlane endeavours to show in his essay, and the discussion has a truly frank and engaging quality. Moreover, it is based upon knowledge and it well deserves careful study. If it deals with hard facts rather than with patriotic enthusiasms this is no reproach, but rather the contrary, for in the end it is the facts which will be the determining element of the situation.

**The Hearts of Man.** By R. M. Wilson, M.B. (London: Henry Frowde and Hodder and Stoughton. 1918. Pp. 182 + xvii. Price 6s. net.)

While, in the popular judgment, man is provided with one heart, and even the physiologist recognises only two, Dr. Wilson has a much more extended vision; he writes without qualification, "there are not two hearts, but five." In this form he crystallises in a phrase his study of the forces which carry on and regulate the circulation of the blood. His argument is that the heart itself is not able to produce all the phenomena to be observed in the circulatory mechanism, and he seeks to demonstrate certain forces which, co-operating with the heart, are of physiological value in securing such a distribution of the blood as is necessary to meet the wants created by the varying activities of the bodily machine. His book is far from being a mere statement of hypotheses and possibilities. On the contrary, the doctrines he advances are based on certain observed facts, as these are noted partly by direct observation and partly by instruments of precision, such as the polygraph. The facts stand, and call for interpretation. Whether Dr. Wilson's interpretations are correct or not is a matter for argument, and a pioneer may not expect a ready following. But they have a stimulating quality and are the outcome of much thought and consideration. Thus it is a pleasure to read his book, and his enterprise deserves recognition and encouragement. As he tells us in his Preface, he has not succeeded in converting either Sir James Mackenzie or Professor Bayliss, and we are bound to say that neither is our own discipleship secure. The hardened reviewer is proverbially stiff-necked; yet he will look with interest for further contributions from Dr. Wilson's studies.

"HOSPITAL SKETCHES."

By Frances Lyndall. (London: George Allen and Unwin, Ltd. Price 2s. net.)

This collection of fourteen sketches, presumably by a nurse, is a slight record of hospital experience, some of which appears to have taken place in France or Italy. They are records rather of the young lady's impressions of her life than of things seen and remembered, and only in "Our Italian Orderly," the best of the series, and in "The Protégé of a Princess" is there any attempt at character studies. There is less humour and more sentiment than will be agreeable to all tastes, but the little book is honest through its limitations, and probably presents a fair picture of the impressions left by a military hospital on the mind of a young V.A.D. nurse. As such it is an index of a large and enthusiastic class which has made its first acquaintance with the rough-and-tumble of the world through nursing during the war.

